# Inhibition of Murine Pulmonary Microvascular Endothelial Cell Apoptosis Promotes Recovery of Barrier Function under Septic Conditions

**DOI:** 10.1155/2017/3415380

**Published:** 2017-01-30

**Authors:** Lefeng Wang, Sanjay Mehta, Michael Brock, Sean E. Gill

**Affiliations:** ^1^Centre for Critical Illness Research, Lawson Health Research Institute, London, ON, Canada; ^2^Department of Medicine, Schulich School of Medicine and Dentistry, Western University, London, ON, Canada; ^3^Division of Respirology, Schulich School of Medicine and Dentistry, Western University, London, ON, Canada; ^4^Department of Physiology and Pharmacology, Schulich School of Medicine and Dentistry, Western University, London, ON, Canada

## Abstract

Sepsis is characterized by injury of the pulmonary microvasculature and the pulmonary microvascular endothelial cells (PMVEC), leading to barrier dysfunction and acute respiratory distress syndrome (ARDS). Our recent work identified a strong correlation between PMVEC apoptosis and microvascular leak in septic mice in vivo, but the specific role of apoptosis in septic PMVEC barrier dysfunction remains unclear. Thus, we hypothesize that* PMVEC apoptosis is likely required for PMVEC barrier dysfunction under septic conditions in vitro*. Septic stimulation (mixture of tumour necrosis factor *α*, interleukin 1*β*, and interferon *γ* [cytomix]) of isolated* murine* PMVEC resulted in a significant loss of barrier function as early as 4 h after stimulation, which persisted until 24 h. PMVEC apoptosis, as reflected by caspase activation, DNA fragmentation, and loss of membrane polarity, was first apparent at 8 h after cytomix. Pretreatment of PMVEC with the pan-caspase inhibitor Q-VD significantly decreased septic PMVEC apoptosis and was associated with reestablishment of PMVEC barrier function at 16 and 24 h after stimulation but had no effect on septic PMVEC barrier dysfunction over the first 8 h. Collectively, our data suggest that early septic murine PMVEC barrier dysfunction driven by proinflammatory cytokines is not mediated through apoptosis, but PMVEC apoptosis contributes to late septic PMVEC barrier dysfunction.

## 1. Introduction

Acute respiratory distress syndrome (ARDS), which has a 30–40% mortality rate, is characterized by severe pulmonary inflammation and high-permeability, proteinaceous edema [1, 2]. Sepsis is the most common cause of ARDS [1, 3–5]. Septic organ dysfunction, including the lung injury present in ARDS, is due in large part to systemic inflammation leading to dysfunction of the microvasculature, especially the microvascular endothelial cells (MVEC) [5–9]. Microvascular dysfunction is characterized by impaired barrier function (increased permeability leading to extravascular leak of protein-rich edema) and neutrophil (PMN) influx into organs [10–14], microvascular thrombosis [15, 16], and impaired distribution of blood flow in microvascular beds [17]. Microvascular dysfunction is clinically important, as it has been documented early in the course of sepsis in humans, and is associated with increased mortality [7, 8], especially if it persists over time [9].

Pulmonary microvascular dysfunction in sepsis and ARDS is principally due to activation, injury, and dysfunction of pulmonary MVEC (PMVEC). Multiple mechanisms promote septic PMVEC dysfunction, including activation by cytokines, mechanical interaction with activated leukocytes, and exposure to harmful leukocyte-derived molecules, such as oxidants (including nitric oxide). These factors result in PMVEC abnormalities, including disruption of inter-PMVEC junctions and cytoskeleton-driven retraction [2, 5, 10–12, 18–21]. Recently, we identified a correlation between PMVEC apoptosis in vivo and increased pulmonary microvascular permeability following cecal ligation and perforation- (CLP-) induced sepsis in mice [22, 23]. Furthermore, we demonstrated that systemic administration of Q-VD, a synthetic inhibitor of caspases, decreased septic PMVEC apoptosis, which was associated with reduced septic pulmonary microvascular permeability [23].

Apoptosis is a highly regulated, energy-dependent, enzymatic process of cell death, important in development and tissue homeostasis, but is also activated under inflammatory/pathologic conditions, such as sepsis. Apoptotic cell death is characterized by activation of cysteine proteases known as caspases, a loss of cell membrane polarization, and fragmentation of the DNA leading to condensed nuclei [24, 25]. Initiation of apoptosis is regulated by multiple pathways, which culminate in final common effector caspase activation. One of these, the extrinsic (or receptor-mediated) pathway depends on signalling by members of the tumour necrosis factor (TNF) cytokine family (i.e., TNF*α*) suggesting that stimulation of PMVEC with a combination of proinflammatory cytokines (e.g., mixture of TNF*α*, interleukin [IL] 1*β*, and interferon [IFN] *γ*) may lead to PMVEC apoptosis [24, 26]. However, while some studies support the ability of proinflammatory cytokines (i.e., TNF*α*) to induce endothelial cell (EC) apoptosis, these cytokines do not consistently induce apoptosis, depending on particular EC type and method of assessment of both EC barrier function and apoptosis (Table 1) [27–42]. Further, it has not been clearly established whether PMVEC apoptosis is a driving factor in early and late septic EC barrier dysfunction or whether apoptosis has a role in recovery from septic injury and repair of the microvascular permeability barrier. Specifically, previous studies, primarily in macrovascular EC such as human umbilical vein EC (HUVEC), suggest that septic EC barrier dysfunction may correlate with EC apoptosis; however, many of these studies do not clearly demonstrate that EC apoptosis causes barrier dysfunction (Table 1). Additionally, there is also evidence that EC leak can occur in the absence of EC apoptosis (Table 1). Our previous work, however, suggests PMVEC apoptosis as a critical mechanism for the loss of PMVEC barrier function in vivo [22, 23]. Thus, we hypothesize that PMVEC apoptosis is likely required for PMVEC barrier dysfunction under septic conditions in vitro. Furthermore, it is likely that PMVEC apoptosis also prevents reestablishment of normal PMVEC barrier function.

## 2. Methods

### 2.1. PMVEC Isolation

PMVEC were isolated from the lungs of C57Bl/6 mice, as previously described [10, 43]. In brief, lungs were isolated, finely minced, and then digested with collagenase before incubation with magnetic microbeads (Dynal Biotech Inc., Lake Success, NY) coupled to anti-CD31 (platelet endothelial cell adhesion molecule, PECAM) antibodies (BD Pharmingen, Franklin Lakes, NJ). Microbead-bound PMVEC were captured, washed, suspended in growth medium (Dulbecco's modified Eagle's medium [DMEM] with 20% Fetal Bovine Serum, 5 mM glucose, 4 mM L-Glutamine, 1 mM Sodium Pyruvate, and Phenol Red; Invitrogen, Carlsbad, CA), and then seeded in gelatin-coated cell-culture flasks. Once approximately 90% confluent, cells were stained with fluorescent acetylated-low density lipoprotein (LDL) (Biomedical Technologies, Stoughton, MA) and assessed by immunofluorescence or stained with antibodies against CD31, CD34, CD146, and CD202b conjugated to pacific blue, phycoerythrin, fluorescein isothiocyanate (FITC), or allophycocyanin, respectively (VWR Scientific Inc., Radnor, PA), and assessed by flow cytometry (easyCyte Guava 12HT; Millipore, Billerica, MA, USA). Collectively, these processes resulted in PMVEC isolates with 99% homogeneity, which were then cultured and used between passages 4 and 8.

### 2.2. Assessment of PMVEC Barrier Integrity

PMVEC barrier function was assessed in vitro by culturing 2.5 × 10^4^ PMVEC on gelatin-coated 24-well cell-culture inserts (3.0 *μ*m pore, VWR) in full DMEM medium as we have done previously [10, 43]. During growth, culture medium was changed every second day and PMVEC monolayer permeability was assessed every second day by measuring transendothelial electrical resistance (TEER; EVOM2 Endothelial Voltohmmeter; World Precision Instruments, Sarasota, FL). Individual PMVEC monolayer TEER was corrected for TEER across an empty insert. A fully intact basal PMVEC permeability barrier was accepted when TEER stabilized (±5%).

PMVEC monolayer permeability under basal/resting and septic conditions (cytomix: equimolar solution of TNF*α*, IL1*β*, and IFN*γ* used to mimic a septic response, 0.3–100 ng/mL, PeproTech, Rocky Hill, NJ) was assessed over a time course (2–24 h) using three techniques: (i) TEER (as above), (ii) FITC-labelled dextran flux (4 kDa), and (iii) EB-labelled albumin flux (67 kDa). The levels of trans-PMVEC macromolecular flux of the smaller molecular weight FITC-labelled dextran (4 kDa) or larger molecular weight EB-labelled albumin (67 kDa) from the upper chamber into the lower chamber of the cell-culture inserts were measured over exactly 60 mins as we have done previously [10, 43]. Briefly, both EB-labelled albumin (bovine serum albumin, 33.5 *μ*g total in 250 *μ*L; Sigma, Oakville, Ontario) and FITC-labelled dextran (125 *μ*g total in 250 *μ*L; Sigma) were added directly to the upper chamber of the cell-culture insert. After 1 h, inserts were removed and the conditioned media from the lower chamber collected. EB-labelled albumin flux was determined by measuring the absorbance of the conditioned medium (620 nm) and comparing to a standard curve of EB-labelled albumin (Victor3 multilabel microplate reader, PerkinElmer, Inc. Waltham, MA, USA). Trans-PMVEC FITC-labelled dextran flux was determined by collecting the lower chamber medium, measuring the fluorescence (excitation peak wavelength: 488 nm and emission peak wavelength: 525 nm), and comparing this to completely equilibrated FITC-dextran in both chambers of control wells (labelled 100%; Victor3 multilabel microplate reader).

Basal and septic PMVEC permeability was also assessed in the presence or absence of the broad-spectrum potent caspase inhibitors carbobenzoxy-valyl-alanyl-aspartyl-[O-methyl]-fluoromethylketone (z-VAD-FMK, 100 *μ*M, BD Biosciences, Mississauga, ON) or quinoline-valyl-aspartyl(Ome)-CH2-O-phenoxy (Q-VD, 50 *μ*M, APExBIO, Boston, MA) [45]. For these studies, inhibitors were administered simultaneously with the septic stimulus (cytomix).

### 2.3. Quantification of PMVEC Apoptosis

To identify features of apoptosis in PMVEC, three different molecular markers were assessed using fluorescence microscopy and flow cytometry under basal and septic conditions: (1) caspase activation, (2) DNA fragmentation, and (3) loss of cell membrane polarity.

To detect caspase activation, PMVEC were stained with the Sulforhodamine (SR) FLICA Poly Caspase Assay Kit as per manufacturer's instructions (Immunohistochemistry Technologies, Bloomington, MN). Briefly, SR FLICA was added to PMVEC culture medium for the final 1 h of stimulation after which PMVEC were fixed with 10% formalin. Hoechst's stain (Hoechst 33342, Life Technologies Inc., Burlington, ON) was then used to fluorescently label PMVEC nuclei. Cells were then imaged using fluorescent microscopy (FLICA excitation/emission: 550 nm/590–600 nm; Hoechst excitation/emission: 361 nm/486 nm). The number of FLICA and Hoechst positive cells per field of view was counted through the use of a macro in ImageJ (National Institutes of Health). Automated counts of positive cells were confirmed with manual counts by two blinded reviewers.

Late-stage apoptotic DNA fragmentation in PMVEC was examined by terminal deoxynucleotidyl transferase dUTP nick end labeling (TUNEL; excitation/emission: 494/521 nm; In Situ Cell Death Detection, Roche, Laval, QC). For these studies, PMVEC were fixed in 10% formalin following basal and septic (cytomix) stimulation and then permeabilized with a 1% Na^+^ citrate/0.1% Triton X-100 solution. Following permeabilization, TUNEL staining was used to identify PMVEC with DNA fragmentation and Hoechst stain was used to label all PMVEC nuclei. Cells were then imaged using fluorescent microscopy and the number of TUNEL and Hoechst positive cells per field of view was determined as above.

Loss of cell membrane polarization (as indicated by presence of cell surface phosphatidylserine) was assessed by staining PMVEC with FITC-conjugated Annexin V and propidium iodide (PI; Invitrogen, Burlington, ON). For these studies, PMVEC were stimulated with PBS or cytomix, lifted by trypsinization, and stained with Annexin V and PI in binding buffer (0.1 M HEPES pH 7.4; 1.4 M NaCl; 25 mM CaCl_2_). The presence of Annexin V and PI staining was then analyzed by flow cytometry (easyCyte Guava 12HT). Annexin V^+^/PI^−^ cells were considered apoptotic cells, whereas Annexin V^+^/PI^+^ cells were considered dead cells and Annexin V^−^/PI^−^ cells were considered live cells.

### 2.4. Quantification of PMVEC Detachment

The degree of PMVEC attachment was assessed under basal and septic conditions and following treatment with Q-VD. Detached cells were quantified by pooling the conditioned media collected from each well with the supernatant from a single wash (PBS) of each well. Following centrifugation at 400 RCF for 10 min at 4°C, the supernatant was removed, the cell pellet resuspended in 0.1% albumin/PBS, and the detached PMVEC cytospun onto slides. Detached PMVEC were then assessed by TUNEL/Hoechst staining as described above.

### 2.5. Statistical Analysis

Data are reported as mean ± SEM and were analyzed using GraphPad Prism 5. Differences between groups were assessed by *t*-tests (one measured variable) or by a two-way ANOVA with Bonferroni post hoc testing (two independent variables). Significance threshold was set at *α* = 0.05 and experiments were replicated at least 3 times.

## 3. Results

### 3.1. Dose Response and Time Course of Cytomix-Induced PMVEC Permeability

**Figure 1:**
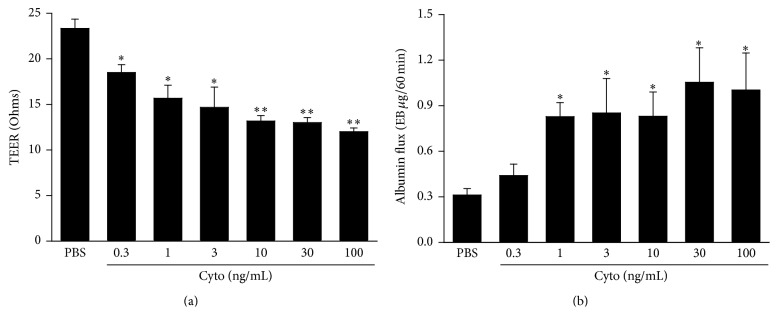
Cytomix induces a dose-dependent increase in mouse PMVEC permeability. PMVEC had significantly higher permeability 4 h after cytomix stimulation by 2 assays: (a) lower transendothelial electrical resistance (TEER) and (b) higher Evans blue- (EB-) labelled albumin flux. Furthermore, by both measures, permeability was maximal at 30 ng/mL cytomix. *∗* and *∗∗* represent *P* < 0.05 and 0.01 compared with PBS (one-way ANOVA), respectively. *n* = 3.

In our previous in vivo and in vitro studies, there is significant septic PMVEC barrier dysfunction at 4 h after septic stimulation [12, 22, 23, 43]. To identify the concentration of cytomix required to induce maximal PMVEC permeability as indicated by two complementary techniques, TEER and EB-albumin flux, PMVEC were treated with a range of cytomix concentrations. Under basal conditions, PMVEC achieved a stable TEER of 23.3 ± 1.0 Ohms (Figure 1(a)). PMVEC TEER was significantly decreased (79.4 ± 0.2% versus PBS) 4 h following stimulation with 0.3 ng/mL cytomix and continued to decrease in a dose-dependent manner until 10 ng/mL cytomix (56.7 ± 4.5% versus PBS; Figure 1(a)). PMVEC permeability to protein, as measured by EB-labelled albumin flux across the PMVEC monolayer, was significantly increased versus baseline following stimulation with 1 ng/mL cytomix (264.5 ± 11.0% versus PBS; Figure 1(b)). PMVEC permeability became maximal at 30 ng/mL of cytomix (338.7 ± 21.9% versus PBS; Figure 1(b)).

**Figure 2:**
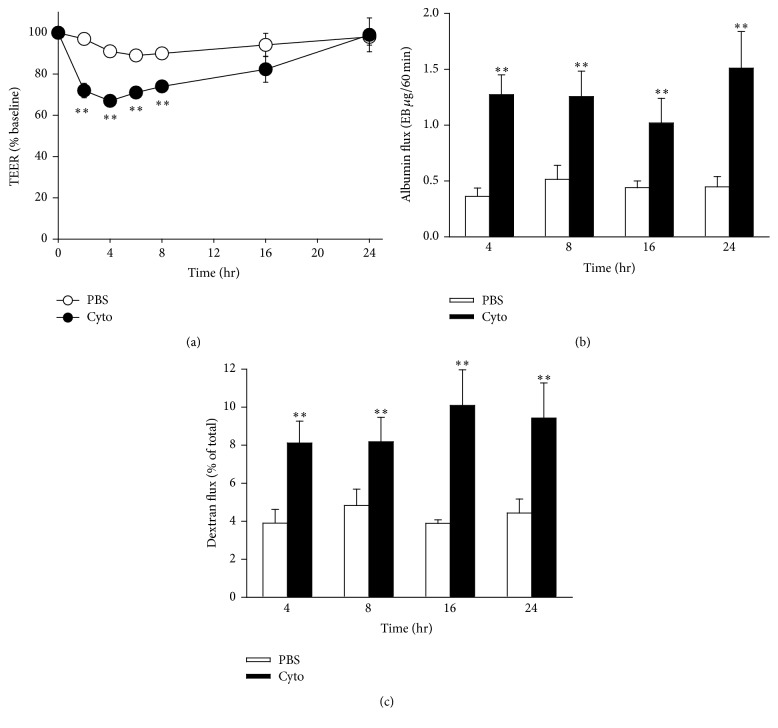
Time course of cytomix-induced mouse PMVEC hyperpermeability. Cytomix-stimulated PMVEC had significantly increased permeability by 4 h after stimulation versus PBS by 3 assays: lower TEER (a) and higher macromolecular flux including fluorescein isothiocyanate- (FITC-) labelled dextran (b) and EB-labelled albumin (c). Interestingly, leak as assessed by TEER appeared to recover by 16–24 h after cytomix, whereas septic enhanced macromolecular flux persisted. *∗∗* represents *P* < 0.01 compared with PBS (two-way ANOVA). *n* = 8.

The time course of septic PMVEC hyperpermeability was more rigorously defined following stimulation with 30 ng/mL cytomix. Following cytomix stimulation, TEER was significantly reduced by 2 h and was maximally reduced by 4 h (Figure 2(a)). After 4 h, TEER gradually recovered returning to baseline by 24 h after cytomix stimulation (Figure 2(a)). PMVEC permeability to small (4 kDa dextran) and large (67 kDa albumin) macromolecules was significantly increased at 4 h after cytomix and remained significantly elevated at 24 h after cytomix with no evidence of recovery (Figures 2(b) and 2(c)).

### 3.2. Time Course of Cytomix-Induced PMVEC Apoptosis

**Figure 3:**
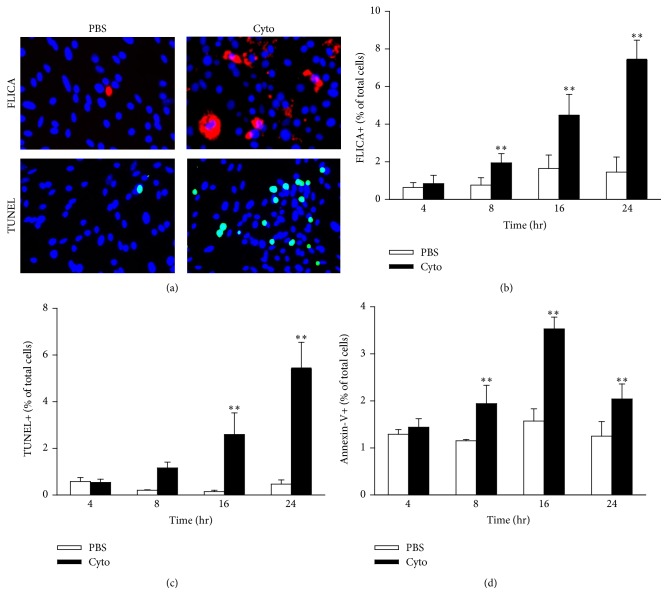
Cytomix induces mouse PMVEC apoptosis. (a) Cytomix stimulation for 24h leads to an increased number of cells stained positive for active caspases (fluorescent inhibitor of caspases [FLICA]; upper row; red) and fragmented DNA (terminal deoxynucleotidyl transferase dUTP nick end labeling [TUNEL]; lower row; green). Nuclei were stained with Hoechst 33342. Quantification revealed significant increases in FLICA+ (b), TUNEL+ (c), and Annexin V+/propidium iodide- (PI-) cells (d) by 8h (FLICA and Annexin V) and 16h (TUNEL) after cytomix. All 3 markers indicated persistent increases in septic PMVEC apoptosis at 24h. ^*∗∗*^*P* < 0.01 compared with PBS (two-way ANOVA). *n* = 5-6.

To begin to assess the role of PMVEC apoptosis in the increased PMVEC permeability following stimulation with cytomix, we examined three different molecular features associated with apoptosis over a time course: caspase activation (FLICA+), loss of cell membrane polarity (Annexin V+), and DNA fragmentation (TUNEL+). PMVEC apoptosis, as evidenced by greater FLICA and Annexin V staining, was significantly increased by 8 h after cytomix stimulation (237.5 ± 26.3% and 172.7 ± 21.1% versus PBS for FLICA and Annexin V, resp.), which persisted until 24 h after cytomix (569.2 ± 14.9% and 153.8 ± 15.0% versus PBS for FLICA and Annexin V, resp.; Figure 3). Similarly, TUNEL staining was significantly increased by 16 h after cytomix stimulation (866.7 ± 34.6% versus PBS) and was still evident at 24 h (900.0 ± 20.4% versus PBS; Figure 3).

**Figure 4:**
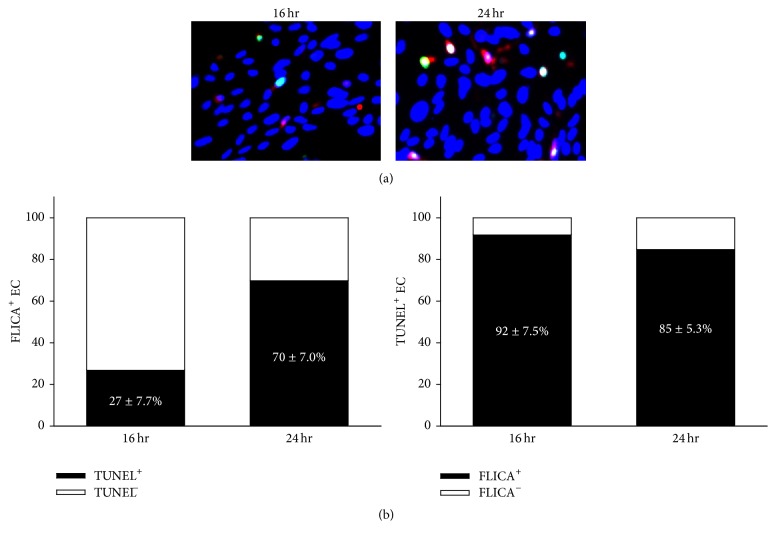
Caspase activation correlates with DNA fragmentation as a marker of PMVEC apoptosis. (a) Cytomix stimulation leads to an increased number of cells stained positive for both active caspases (FLICA; red) and fragmented DNA (TUNEL; green). Note that overlap between the 2 markers of apoptosis appears as yellow-white. Nuclei were stained with Hoechst 33342. (b) Quantitation of double-positive cells revealed that at 16 h, only 27 ± 7.7% of FLICA+ cells were also TUNEL+, whereas by 24 h, this number had increased to 70 ± 7.0%. Interestingly, almost all TUNEL+ cells were FLICA+ at both 16 h (92 ± 7.5%) and 24 h (85 ± 5.3%). *n* = 4.

Caspase activation [FLICA] is considered an early marker of apoptosis whereas DNA fragmentation [TUNEL] is considered a late-stage marker of apoptosis. Examination of PMVEC double stained with FLICA and TUNEL demonstrated that at 16 h afer cytomix, 27.4 ± 7.7% of FLICA+ cells were TUNEL+, and by 24 h after cytomix, the percentage of FLICA+ cells that were also TUNEL+ increased to 70.6 ± 7.0% (Figure 4). Most of the TUNEL+ cells were also FLICA+ at both 16 and 24 h after cytomix (Figure 4).

### 3.3. Effect of Caspase Inhibition on Cytomix-Induced PMVEC Leak and Apoptosis

**Figure 5:**
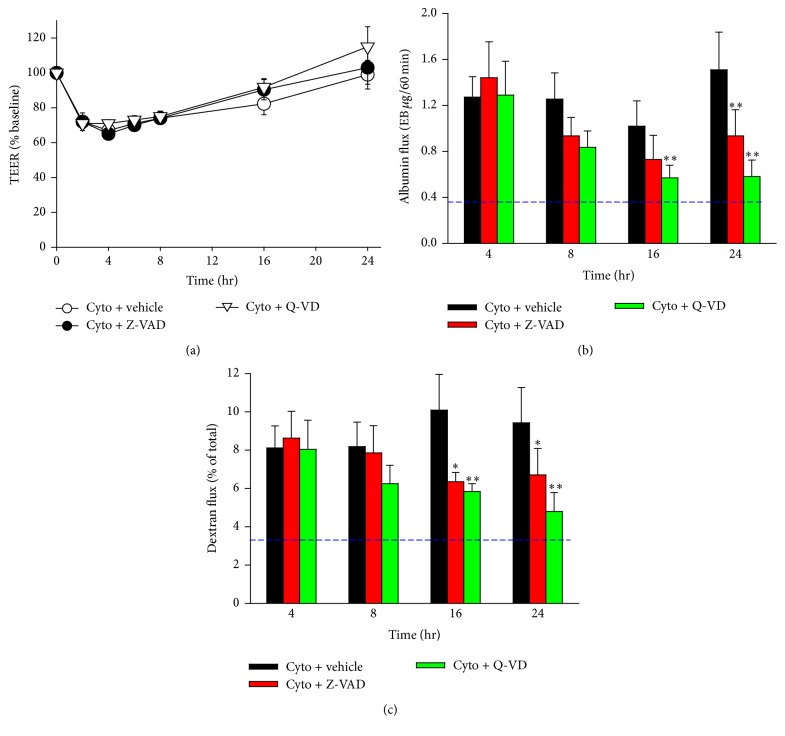
Caspase activity contributes to persistent septic PMVEC macromolecular hyperpermeability. (a) Inhibition of caspase activity (Z-VAD, 100 *μ*M; Q-VD, 50 *μ*M) following cytomix stimulation did not appear to affect mouse PMVEC TEER at any of the time points examined versus vehicle treatment (dimethyl sulfoxide [DMSO]). In contrast, septic increases in macromolecular flux across PMVEC, including EB-albumin (b) and FITC-dextran (c), were significantly attenuated at 16 h and 24 h after cytomix by inhibition of caspase activity. Dashed lines indicate average basal level (treated with vehicle alone). Of note, inhibition of caspase activity had no effect on septic PMVEC increases in macromolecular flux at earlier time points (4 h and 8 h). *∗* and *∗∗* represent *P* < 0.05 and 0.01 compared with PBS, respectively (two-way ANOVA). *n* = 6–8.

To determine the contribution of PMVEC apoptosis to cytomix-induced loss of PMVEC barrier function, PMVEC were treated with two broad-spectrum caspase inhibitors, Z-VAD and Q-VD. Treatment with either Z-VAD or Q-VD had no effect on cytomix-induced decreases in PMVEC TEER compared to cytomix alone (Figure 5(a)). Similarly, no significant effects of Z-VAD or Q-VD were observed on cytomix-induced macromolecular flux (dextran and albumin) at 4 h and 8 h after cytomix (Figures 5(b) and 5(c)). Treatment with Z-VAD, however, significantly reduced cytomix-induced dextran flux versus cytomix alone at both 16 and 24 h and significantly reduced cytomix-induced albumin flux versus cytomix alone at 24 h (Figures 5(b) and 5(c)). Furthermore, treatment with Q-VD resulted in a significant reduction in both dextran and albumin flux at 16 and 24 h after cytomix compared to cytomix alone (Figures 5(b) and 5(c)).

**Figure 6:**
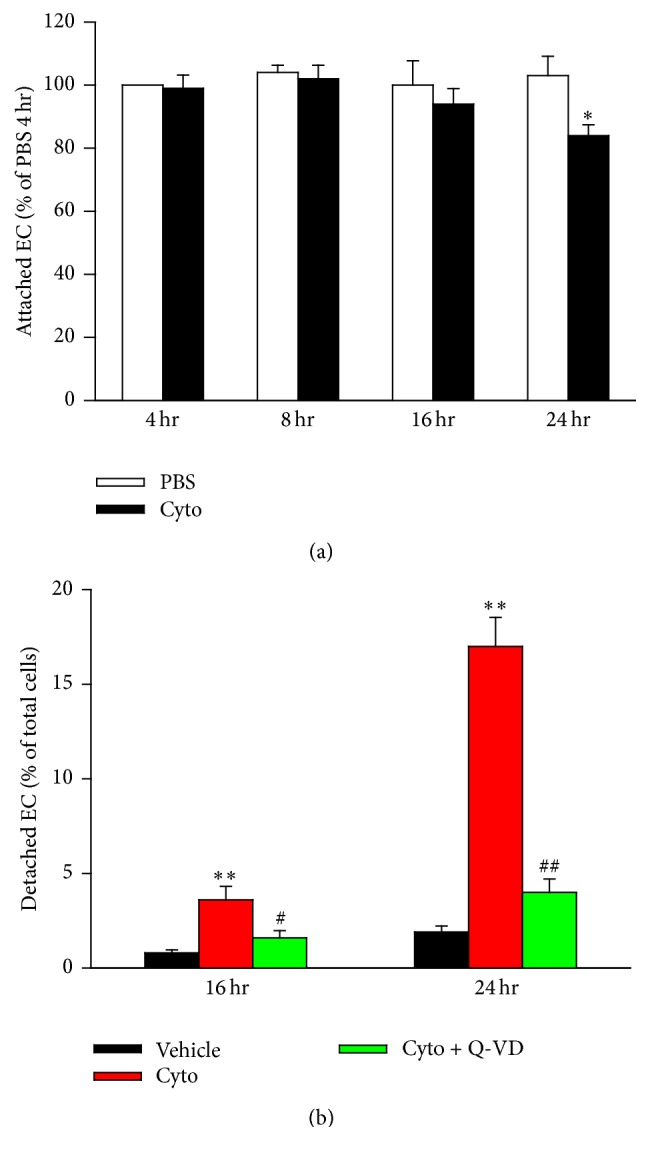
Inhibition of caspase activity reduces cell detachment. (a) Stimulation with cytomix significantly decreases the number of attached mouse PMVEC after 24 h. ^*∗*^*P* < 0.05 compared with PBS (two-way ANOVA). *n* = 4–6. (b) Treatment of cytomix-stimulated PMVEC with Q-VD (50 *μ*M) significantly increased the number of attached cells versus vehicle-treated. ^*∗∗*^*P* < 0.01 compared with vehicle (DMSO); # and ## represent *P* < 0.05 and 0.01 compared with cytomix-treated group, respectively (two-way ANOVA).

Apoptosis is thought to be associated with an increase in detached cells [44]. Assessment of PMVEC detachment following cytomix stimulation revealed a significant reduction in cell attachment following 24 h of cytomix stimulation (Figure 6(a)). Furthermore, cytomix stimulation was also associated with a significant increase in the percentage of detached PMVEC at 16 h and 24 h following cytomix stimulation (360.0 ± 19.4% and 894.7 ± 8.8% versus PBS, resp.; Figure 6(b)). Importantly, treatment with Q-VD was found to significantly reduce the percentage of detached PMVEC at both 16 h (160.0 ± 23.1% versus PBS) and 24 h (210.5 ± 17.5% versus PBS) after cytomix (Figure 6(b)).

**Figure 7:**
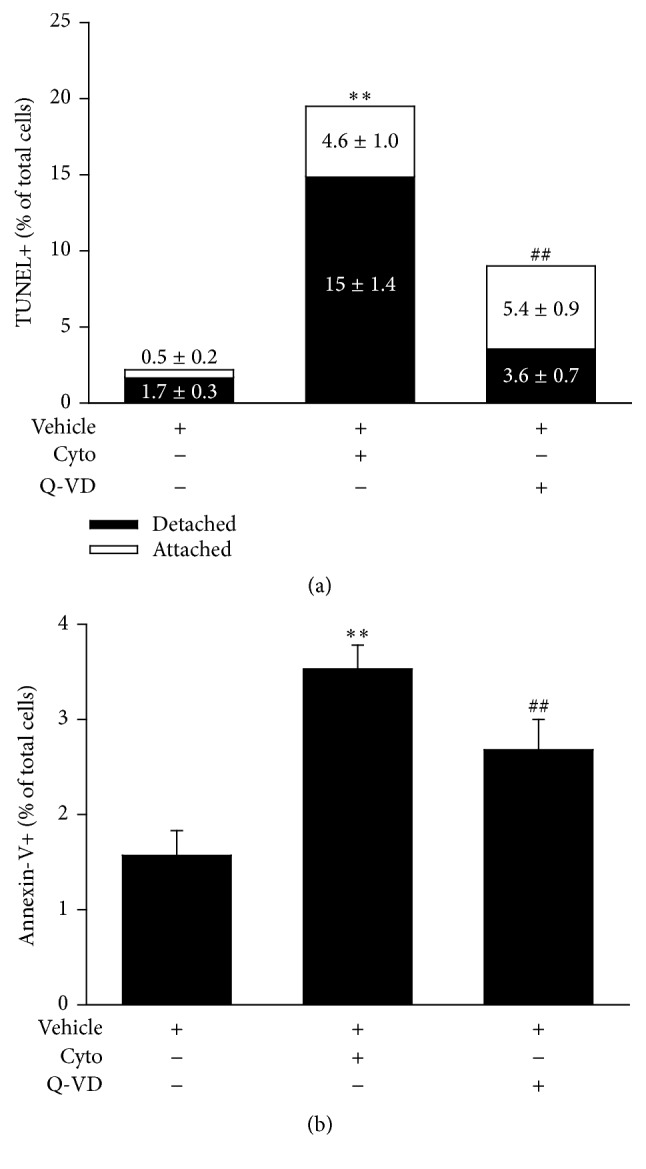
Inhibition of caspase activity reduces PMVEC apoptosis. (a) Stimulation of PMVEC with cytomix (24 h) led to a significant increase in TUNEL+ cells and this increase was significantly reduced by treatment with Q-VD (50 *μ*M). *n* = 6. (b) Flow cytometric analysis revealed an increased number of early apoptotic PMVEC (Annexin V+/PI−) following cytomix stimulation (16 h) that was significantly reduced by treatment with Q-VD (50 *μ*M). *n* = 4. *∗∗* or ## represents *P* < 0.01 compared with vehicle or cytomix group, respectively (one-way ANOVA).

Apoptosis was then examined in Q-VD treated PMVEC to confirm that the observed decreases in cytomix-induced macromolecular flux and PMVEC detachment were due to decreases in apoptotic PMVEC death. As previously observed (Figure 3(c)), the percentage of TUNEL+ cells was significantly increased at 24 h after cytomix (890.9 ± 12.2% versus PBS); however, treatment with Q-VD resulted in a significant reduction in the percentage of TUNEL+ PMVEC following cytomix stimulation (409.1 ± 17.8% versus PBS; Figure 7(a)). Interestingly, inclusion of detached cells in the assessment of apoptosis resulted in an increase in the percentage of TUNEL+ PMVEC compared with assessment in attached cells alone (19.6 ± 2.4% versus 4.6 ± 1.0% resp.; Figure 7(a)). Similar to TUNEL+ PMVEC, the percentage of Annexin V+ PMVEC was significantly increased following cytomix stimulation (versus vehicle control), and treatment with Q-VD was associated with a significant reduction in the percentage of Annexin V+ PMVEC versus cytomix-stimulated PMVEC in the absence of Q-VD (Figure 7(b)).

## 4. Discussion

In the current report, we studied an in vitro model of septic ARDS by isolating, culturing, and studying murine PMVEC in vitro under septic conditions induced by exposure to multiple sepsis-relevant proinflammatory cytokines. Our current work confirms that stimulation of PMVEC with this mixture of 3 clinically relevant cytokines leads to a dose-dependent increase in PMVEC permeability over a biphasic time course, including acute 4–8 h barrier dysfunction characterized by both reduced trans-PMVEC electrical resistance (TEER) and enhanced macromolecular permeability and late-phase 16–24 h persistence of this septic PMVEC macromolecule hyperpermeability despite recovery of TEER to baseline. Septic PMVEC apoptosis was documented using 3 independent and complementary markers and was found to be significant as early as 8 h and persisted at 16–24 h after cytomix stimulation. Early septic PMVEC hyperpermeability was not apoptosis-dependent; however, delayed PMVEC barrier dysfunction was abrogated following effective inhibition of apoptosis using two distinct caspase inhibitors, coincident with markedly inhibited PMVEC apoptosis and PMVEC detachment.

In sepsis, multiple organ dysfunction, including ARDS, is presumed due to systemic inflammatory injury of the microvasculature, especially the MVEC [5–9]. There is evidence for microvascular and MVEC dysfunction and injury in human sepsis. For example, microvascular dysfunction has been documented early in the course of human sepsis [7, 8, 46, 47]. In addition, increased numbers of circulating EC and soluble markers of EC damage (e.g., intercellular adhesion molecule 1, von Willebrand factor [vWF], and vascular endothelial growth factor receptor 1) correlate with more severe sepsis and higher mortality [48–54]. Furthermore, this septic microvascular dysfunction is clinically relevant as the presence of microvascular dysfunction in human sepsis is associated with more severe sepsis, organ dysfunction, and increased mortality [7, 8, 46]. Moreover, clinical outcomes including survival were especially poor if septic microvascular dysfunction persisted over time despite usual clinical management [9].

Specifically, in ARDS, there are similar although more limited data to support pulmonary microvascular and MVEC injury and dysfunction. For example, pulmonary microvascular dysfunction, as reflected by higher measured ventilatory dead space was found to be associated with more severe ARDS and greater mortality [55]. Similarly, the presence of pulmonary vascular disease manifesting as pulmonary hypertension in patients with ARDS is an independent marker of poor prognosis [56]. In addition, elevated soluble plasma levels of several EC-derived proteins suggestive of more severe EC injury, including angiopoetin-2, thrombomodulin, and vWF in ARDS patients, are associated with higher mortality [57–60]. Specifically, for Ang-2, elevated levels are associated with a greater incidence of ARDS in patients at risk [57], and increasing levels over the first few days of infection-associated ARDS are more predictive of higher mortality than baseline levels [58]. Conceptually, pulmonary microvascular injury and dysfunction are thought to be central to indirect causes of ARDS (e.g., sepsis) and are also likely required for the development of ARDS in patients with clinical conditions characterized by direct lung insults (e.g., pneumonia and aspiration).

This septic microvascular and MVEC dysfunction is especially characterized by impaired barrier function, with the septic hyperpermeability resulting in protein-rich tissue edema and PMN influx into organs. Clearly, leak of protein-rich fluid from the pulmonary microvasculature into the interstitial and alveolar spaces is one of the defining pathophysiological features of ARDS [61, 62] and of animal models of sepsis-induced lung injury [11, 12, 22, 23]. Although many studies have advanced our understanding of the mechanisms regulating PMVEC barrier dysfunction in ARDS, many of these reports examined barrier function in either EC from the macrovasculature (e.g., HUVEC and pulmonary artery endothelial cells [PAEC]) or EC from systemic vascular beds (i.e., brain MVEC and corneal EC) [27–29, 33, 63–66]. While these studies provide insight into potential mechanisms, EC from the micro- and macrovasculature have different biological properties [67–71]. Moreover, the responses of EC from different vascular beds to proinflammatory cytokines vary markedly, especially with respect to apoptosis and the association of apoptosis with increased EC permeability (Table 1) [27–29, 33, 63–66]. Thus, given the importance of the pulmonary microvasculature in sepsis-associated ARDS, our current work focuses specifically on the effect of proinflammatory cytokines on PMVEC.

Multiple mechanisms of PMVEC injury in sepsis and in ARDS have been postulated. These include the actions of cytokines and other soluble circulating molecules, mechanical interaction with activated leukocytes and platelets, and paracrine exposure to injurious molecules released by these cells [2, 11, 12, 18, 19]. Ultimately, these exposures contribute to pulmonary microvascular, specifically PMVEC, injury, dysfunction, and possibly death/apoptosis [72]. EC apoptosis and resulting microvascular permeability are commonly accepted to be pathophysiologically associated, although the direct evidence in support of this relationship is limited [22, 23, 72–74]. For example, our previous work found that septic pulmonary microvascular barrier dysfunction in vivo in mice following CLP-sepsis was associated with increased PMVEC apoptosis and, moreover, that inhibition of apoptosis, via treatment of these mice with Q-VD (a synthetic caspase inhibitor), significantly reduced septic pulmonary microvascular permeability [22, 23]. Additional studies have demonstrated that inhibition of apoptosis following CLP-induced sepsis through treatment with siRNA against caspases or FAS-associated death domain (FADD) rescues septic EC dysfunction, including reducing septic hyperpermeability [72–74]. Furthermore, assessment of apoptosis in vivo revealed the presence of apoptotic EC early in the time course of sepsis (4 h) as well as much later (24 h) depending on the vascular bed studied [23, 73, 74].

Despite the in vivo evidence, however, the role of apoptosis in mediating septic impaired MVEC barrier function, and hence, the pulmonary edema characteristic of ARDS, remains unclear. Multiple studies have attempted to address this question utilizing in vitro models of septic conditions in various EC types (Table 1) [27–39]. Overall, these studies demonstrated that EC stimulation with proinflammatory cytokines sometimes led to the induction of apoptosis, depending on dose, timing, and exact combination of cytokines. However, this EC apoptosis was in many cases only defined by a single marker (e.g., TUNEL), which is a serious limitation as all putative markers of apoptosis can also be observed in other nonapoptotic death mechanisms [75, 76]. Moreover, it is now widely accepted that apoptosis must be supported by a panel of multiple complementary assays, including loss of cell membrane polarization, caspase activation, and DNA fragmentation [75, 76]. Thus, the present study provides a comprehensive assessment of apoptotic cell death (3 complementary markers) over a time course clearly identifying the progressive induction of apoptosis beginning at 8 h and increasing to 24 h after cytomix.

The connection between MVEC apoptosis and barrier dysfunction has also not been firmly established and depends on the EC type, stimulation conditions, and time course with many of the studies utilizing macrovascular EC (e.g., HUVEC) and a single time point (Table 1). For example, stimulation of brain MVEC with TNF*α* and IFN*γ* for 24 h results in apoptosis that is associated with increased brain MVEC barrier dysfunction [33]. Treatment with a caspase inhibitor (Z-VAD), however, only partially restored barrier function. Additionally, stimulation of macrovascular PAEC with TNF*α* resulted in apoptosis as early as 4 h after stimulation that persisted at 20 h [29]. While this apoptosis was also associated with increased permeability across the PAEC monolayer, treatment with Z-VAD did not rescue the enhanced permeability at any time point [29]. It should be emphasized that the study of macrovascular EC (e.g., HUVEC and PAEC) is not biologically relevant to the study of septic microvascular/MVEC dysfunction resulting in organ dysfunction (e.g., ARDS), as MVEC are genotypically and phenotypically very distinct from macrovascular EC [67, 68]. Further, to date, there have been no studies specifically using mouse PMVEC to assess the connection between septic MVEC apoptosis and barrier dysfunction (Table 1). This is critical as septic PMVEC dysfunction is pathobiologically responsible for septic acute lung injury in mice, and mice are one of the primary models currently used for ARDS research due to the ease of genetic manipulation [1, 77]. Thus, our examination of mouse PMVEC apoptosis over a time course clearly establishes for the first time that early septic cytomix-induced murine PMVEC barrier dysfunction is not mediated through PMVEC apoptosis.

There are multiple mechanisms through which loss of PMVEC barrier function has been found to occur; thus, it is not surprising that we found increased PMVEC permeability after 4 h of cytomix stimulation with no evidence of apoptosis. For example, TNF*α* has previously been found to drive loss of corneal EC barrier function through activation of p38 mitogen-activated protein (MAP) kinase and subsequent disassembly of microtubules, as well as adherens and tight junctions [66]. Additionally, examination of barrier function in mouse renal EC following stimulation with TNF*α* demonstrated that increased permeability to albumin was associated with altered actin cytoskeleton, as well as formation of gaps between previously confluent cells and a loss of tight junctions and the EC glycocalyx [78]. Furthermore, inhibition of Rho-associated kinase and myosin light chain kinase, but not inhibition of caspases, rescued the increased permeability as well as the loss of EC glycocalyx and tight junctions [78]. Thus, our data supports these previous studies demonstrating that early cytokine-induced leak across PMVEC is independent of apoptosis; however, it also expands on these studies by clearly demonstrating that the early apoptosis-independent leak transitions to apoptosis-dependent leak as it persists over time.

Loss of EC through loss of cell-extracellular matrix (ECM) interactions and increased EC detachment has long been thought to be involved in loss of barrier function [79]. The increased apoptosis we observed at 16 and 24 h after cytomix stimulation was found to be clearly associated with increased PMVEC detachment. This finding is supported by previous studies that found increased cell detachment following LPS and oxyhemoglobin stimulation of PAEC and brain MVEC, respectively [27, 64]. Similar to our findings, the increased cell detachment in these previous studies was also rescued by inhibition of caspases [27, 64]. Furthermore, the LPS-induced cell detachment was associated with caspase-dependent cleavage of *α*- and *β*-catenin as well as focal adhesion kinase (FAK), critical proteins involved in cell-cell and cell-ECM interactions, respectively. However, the inhibition of caspase activity, which rescued cell detachment, only prevented degradation of proteins involved in cell-ECM interactions, not cell-cell interactions, and unlike our study did not rescue the LPS-induced leak [27]. Together, these studies suggest that the increased cell detachment observed in our study may be due to loss of cell-ECM interactions. However, this remains to be determined. Furthermore, as the rescue of PMVEC detachment observed in our study was also associated with restored PMVEC barrier function, it is possible that additional mechanisms, such as altered cell-cell interactions, are involved.

Our data also demonstrates the importance of the method of assessment of EC barrier function, specifically the importance of using multiple methods to assess EC barrier function, which is not common practice (Table 1). Measurements of TEER versus macromolecular permeability reflect different aspects of EC barrier function and, not surprisingly, could respond differently to inflammatory stimulation. Specifically, the clinically relevant EB-albumin flux is a marker of paracellular and transcellular permeability to large molecules and TEER assesses permeability to charged ions [43]. The acute cytomix-induced PMVEC barrier dysfunction was consistent between TEER and both FITC-dextran and EB-albumin techniques, but persistent septic PMVEC hyperpermeability to macromolecules at later time points was divergent from the observed recovery in TEER. In addition, the acute septic cytokine-induced barrier dysfunction (both decreased TEER and increased macromolecular flux) was not rescued by caspase inhibition (Z-VAD or Q-VD), similar to previously reported studies with PAEC and corneal EC [29, 66]. However, delayed septic hyperpermeability to macromolecules at 16–24 h was rescued by treatment with Z-VAD and Q-VD. Thus, the effect of caspase (apoptosis) inhibition on persistent macromolecular flux across septic PMVEC in the absence of any effect on TEER suggests that barrier function to large molecules was enhanced by caspase inhibition, likely through increased PMVEC attachment, but that PMVEC barrier function was still impaired allowing the passage of small charged ions.

Our findings of persistent septic PMVEC barrier dysfunction at delayed time points are clinically relevant. In ARDS patients, once pulmonary microvascular injury and dysfunction are established, repair of the pulmonary alveolocapillary microvascular EC lining would be necessary and clinically important. Indeed, patients with a greater number of circulating bone-marrow derived endothelial progenitor cells (EPCs), postulated to contribute to PMVEC repopulation following loss of these cells, had better clinical outcomes, including more ventilator-free days and decreased mortality [80]. Similar resident EPC populations have also been identified in the pulmonary microvasculature [81], although their direct involvement in repair of the pulmonary alveolocapillary microvascular EC lining has not been established.

In ARDS, more prolonged clinical illness is associated with a greater need for more intensive and prolonged respiratory support, including mechanical ventilation, higher PEEP levels, and FiO2, which are associated with worse clinical outcomes, specifically greater risk of ventilator-associated pneumonia, higher rates of multisystem organ injury and dysfunction, and greater mortality [82]. As such, ongoing pulmonary microvascular alveolocapillary septic hyperpermeability and persistent high-protein interstitial and alveolar pulmonary edema would contribute to the need for more prolonged respiratory support. Future experiments to define the potential role of human PMVEC apoptosis in septic PMVEC hyperpermeability and septic ARDS, especially the presence of a similar delayed reconstitution of the pulmonary microvascular alveolocapillary permeability barrier, may suggest new therapeutic approaches to promote recovery of patients from ARDS.

Finally, we recognize that our study has limitations. Our previous work found evidence of an association between PMVEC apoptosis and septic pulmonary microvascular permeability in vivo [22, 23]. The findings of our current study, however, suggest that the initial PMVEC barrier dysfunction following stimulation with cytokines is not dependent on apoptosis. This discrepancy is likely due to inherent differences between the natures of these studies. For example, in vivo there are many different cell types, such as pericytes and circulating inflammatory cells, which interact with PMVEC, as well as a complete interstitial ECM and the presence of the glycocalyx on the surface of the PMVEC, all of which are missing or limited in the in vitro setting [83–87]. Furthermore, our in vitro model employed PMVEC cultured alone stimulated with a mixture of three sepsis-relevant cytokines, which is still a less robust septic stimulus than EC would face in vivo (activated leukocytes and bacterial products), as well as the potentially injurious effects of shear stresses associated with blood flow present in the in vivo scenario. However, use of this simplified in vitro isolated PMVEC model as well as the comprehensive assessment of apoptosis (use of three different markers) and PMVEC permeability (use of three complementary measures) allowed for the examination of the function of specifically PMVEC over a comprehensive time course and thereby the identification of the novel potential role of apoptosis-inhibition in reestablishing the PMVEC barrier following septic injury. Future directions of our work will include assessment of apoptosis in more complex models (i.e., PMVEC-PMN coculture) as well as with cells isolated from humans to ensure clinical relevance.

In conclusion, our current data suggests for the first time using mouse cells that early septic PMVEC barrier dysfunction is independent of apoptosis but that persistent septic macromolecule leak is due to loss of adherent cells due to apoptosis, and when apoptosis (specifically caspase activity) is inhibited, PMVEC detachment is decreased, permitting restoration of the normal PMVEC permeability barrier.

## Figures and Tables

**Table 1:** Review of literature on relationship of endothelial cell apoptosis and barrier dysfunction in vitro.

Citation	Species	EC type	EC identification method	Septic treatment	Markers of apoptosis	Measure of trans-EC leak	Leak-apoptosis relationship	Comments
Bannerman et al. 1998 [27]	Bovine	Commercial PAEC cell line	—	LPS	DNA laddering; TUNEL	14C-albumin	+/−	Similar time course of leak and apoptosis; apoptosis inhibition (zVAD) did not affect leak
Petrache et al. 2003 [28]	Bovine	Commercial PAEC cell line	—	TNF*α*	Annexin V; DNA laddering; cleaved caspase 8	TEER	+/−	Apoptosis inhibition (zVAD) treatment inhibited leak; MLCK inhibition reduced apoptosis but did not affect leak
Petrache et al. 2001 [29]	Human	Commercial PAEC	—	TNF*α*	Nucleosome ELISA	TEER	No	No apoptosis observed
Liu et al. 2005 [30]	Human	Commercial HUVEC	—	LPS	DNA fragmentation; Annexin V	EB-albumin	+/−	Time-dependent relationship: leak at all time points, apoptosis only at later time points
Seynhaeve et al. 2006 [31]	Human	Primary HUVEC	Morphology; CD31	TNF*α*, IL1*β*, IFN*γ*	Annexin V; YO-PRO-1	FITC-albumin	+/−	Variable leak-apoptosis relationship depending on combinations of different cytokines
Cardoso et al. 2012 [32]	Rat	Primary brain MVEC	None	LPS	Nuclear morphology; caspase 3 activity	TEER; fluorescein	+/−	Leak-apoptosis correlation following LPS + other inflammatory stimuli, not LPS alone
Lopez-Ramirez et al. 2012 [33]	Human	Commercial brain MVEC	—	TNF*α*, IFN*γ*	Annexin V; caspase 3/7 activity; TUNEL	TEER, FITC-dextran	+/−	Apoptosis inhibition (specific caspase inhibitors) only partially rescued leak; apoptosis only assessed at single time point
Abdullah and Bayraktutan 2014 [34]	Human	Commercial brain MVEC	—	TNF*α*	TUNEL; caspase 3/7 activity	TEER; EB-albumin	+/−	Leak and apoptosis early; leak recovers at later time points but apoptosis increases
Bechelli et al. 2015 [35]	Human	Commercial dermal MVEC cell line	—	*Rickettsia conorii*	Annexin V	TEER	No	Leak occurs before apoptosis; markers of other types of cell death present
Yang et al. 2015 [36]	Human	Commercial pulmonary MVEC	—	LPS	Annexin V	FITC-dextran; FITC-albumin	+/−	Association at a single time point; some conditions had different effects on apoptosis and leak
Wagner et al. 2016 [37]	Human	Commercial HUVEC	—	TNF*α*; procalcitonin	Annexin V	FITC-dextran	+/−	Leak early at low dose; apoptosis present later at high dose
McDonnell et al. 2016 [38]	Human	Primary AoEC	None	*Staphylococcus aureus*	Annexin V	FITC-dextran	Yes	Association at only a single time point
Zhu et al. 2016 [39]	Human	Commercial HUVEC	—	TNF*α* and CXCL10	Cleaved caspase 3	TEER	Yes	Association at only a single time point
Wang et al. 2017 *(present study)*	Mouse	Primary pulmonary MVEC	CD31, CD34, CD176, CD202b	TNF*α*, IL1*β*, IFN*γ*	Annexin V; FLICA (pan-caspase activity); TUNEL	TEER, EB-albumin	Yes	Time-dependent relationship: early leak apoptosis-independent; delayed leak apoptosis-dependent

Aortic endothelial cells, AoEC; C-X-C motif chemokine 10, CXCL10; Evans blue dye, EB; endothelial cell, EC; enzyme-linked immunosorbent assay, ELISA; fluorescein isothiocyanate, FITC; human umbilical vein endothelial cell, HUVEC; interferon gamma, IFN*γ*; interleukin 1 beta, IL1*β*; lipopolysaccharide, LPS; microvascular endothelial cell, MVEC; myosin light-chain kinase, MLCK; pulmonary artery endothelial cells, PAEC; transendothelial electrical resistance, TEER; tumour necrosis factor alpha, TNF*α*; terminal deoxynucleotidyl transferase dUTP nick end labeling, TUNEL; carbobenzoxy-valyl-alanyl-aspartyl-O-methyl, zVAD; +/−, inconsistent/variable association.

## References

[1] Wheeler A. P., Bernard G. R. (2007). Acute lung injury and the acute respiratory distress syndrome: a clinical review. *The Lancet*.

[2] Groeneveld A. B. J. (2002). Vascular pharmacology of acute lung injury and acute respiratory distress syndrome. *Vascular Pharmacology*.

[3] Husak L., Marcuzzi A., Herring J. (2010). National analysis of sepsis hospitalizations and factors contributing to sepsis in-hospital mortality in Canada. *Healthcare quarterly*.

[4] Angus D. C., Linde-Zwirble W. T., Lidicker J., Clermont G., Carcillo J., Pinsky M. R. (2001). Epidemiology of severe sepsis in the United States: analysis of incidence, outcome, and associated costs of care. *Critical Care Medicine*.

[5] Hotchkiss R. S., Karl I. E. (2003). The pathophysiology and treatment of sepsis. *New England Journal of Medicine*.

[6] Glauser M. P. (2000). Pathophysiologic basis of sepsis: considerations for future strategies of intervention. *Critical Care Medicine*.

[7] De Backer D., Creteur J., Preiser J.-C., Dubois M.-J., Vincent J.-L. (2002). Microvascular blood flow is altered in patients with sepsis. *American Journal of Respiratory and Critical Care Medicine*.

[8] Trzeciak S., Dellinger R. P., Parrillo J. E. (2007). Early microcirculatory perfusion derangements in patients with severe sepsis and septic shock: relationship to hemodynamics, oxygen transport, and survival. *Annals of Emergency Medicine*.

[9] Sakr Y., Dubois M.-J., De Backer D., Creteur J., Vincent J.-L. (2004). Persistent-microcirculatory alterations are associated with organ failure and death in patients with septic shock. *Critical Care Medicine*.

[10] Razavi H. M., Wang L. F., Weicker S. (2004). Pulmonary neutrophil infiltration in murine sepsis: role of inducible nitric oxide synthase. *American Journal of Respiratory and Critical Care Medicine*.

[11] Wang L., Taneja R., Razavi H. M., Law C., Gillis C., Mehta S. (2012). Specific role of neutrophil inducible nitric oxide synthase in murine sepsis-induced lung injury in vivo. *Shock*.

[12] Wang L. F., Patel M., Razavi H. M. (2002). Role of inducible Nitric Oxide Synthase in pulmonary microvascular protein leak in murine sepsis. *American Journal of Respiratory and Critical Care Medicine*.

[13] Wagner J. G., Roth R. A. (1999). Neutrophil migration during endotoxemia. *Journal of Leukocyte Biology*.

[14] Granger D. N., Kubes P. (1994). The microcirculation and inflammation: modulation of leukocyte-endothelial cell adhesion. *Journal of Leukocyte Biology*.

[15] Tyml K. (2011). Critical role for oxidative stress, platelets, and coagulation in capillary blood flow impairment in sepsis. *Microcirculation*.

[16] Semeraro N., Ammollo C. T., Semeraro F., Colucci M. (2012). Sepsis, thrombosis and organ dysfunction. *Thrombosis Research*.

[17] Lam C., Tyml K., Martin C., Sibbald W. (1994). Microvascular perfusion is impaired in a rat model of normotensive sepsis. *Journal of Clinical Investigation*.

[18] Farley K. S., Wang L. F., Razavi H. M. (2006). Effects of macrophage inducible nitric oxide synthase in murine septic lung injury. *American Journal of Physiology - Lung Cellular and Molecular Physiology*.

[19] Handa O., Stephen J., Cepinskas G. (2008). Role of endothelial nitric oxide synthase-derived nitric oxide in activation and dysfunction of cerebrovascular endothelial cells during early onsets of sepsis. *American Journal of Physiology—Heart and Circulatory Physiology*.

[20] Dejana E. (1996). Endothelial adherens junctions. Implications in the control of vascular permeability and angiogenesis. *Journal of Clinical Investigation*.

[21] Shelton J. L., Wang L., Cepinskas G., Inculet R., Mehta S. (2008). Human neutrophil-pulmonary microvascular endothelial cell interactions in vitro: differential effects of nitric oxide vs. peroxynitrite. *Microvascular Research*.

[22] Gill S. E., Taneja R., Rohan M., Wang L., Mehta S. (2014). Pulmonary microvascular albumin leak is associated with endothelial cell death in murine sepsis-induced lung injury in vivo. *PLoS ONE*.

[23] Gill S. E., Rohan M., Mehta S. (2015). Role of pulmonary microvascular endothelial cell apoptosis in murine sepsis-induced lung injury in vivo. *Respiratory Research*.

[24] Martin T. R. (2005). Apoptosis and epithelial injury in the lungs. *Proceedings of the American Thoracic Society*.

[25] Riedl S. J., Salvesen G. S. (2007). The apoptosome: signalling platform of cell death. *Nature Reviews Molecular Cell Biology*.

[26] Drakopanagiotakis F., Xifteri A., Polychronopoulos V., Bouros D. (2008). Apoptosis in lung injury and fibrosis. *European Respiratory Journal*.

[27] Bannerman D. D., Sathyamoorthy M., Goldblum S. E. (1998). Bacterial lipopolysaccharide disrupts endothelial monolayer integrity and survival signaling events through caspase cleavage of adherens junction proteins. *Journal of Biological Chemistry*.

[28] Petrache I., Birukov K., Zaiman A. L. (2003). Caspase-dependent cleavage of myosin light chain kinase (MLCK) is involved in TNF-*α*-mediated bovine pulmonary endothelial cell apoptosis. *FASEB Journal*.

[29] Petrache I., Verin A. D., Crow M. T., Birukova A., Liu F., Garcia J. G. N. (2001). Differential effect of MLC kinase in TNF-*α*-induced endothelial cell apoptosis and barrier dysfunction. *American Journal of Physiology—Lung Cellular and Molecular Physiology*.

[30] Liu D., Zhang D., Scafidi J., Wu X., Cramer C. C., Davis A. E. (2005). C1 inhibitor prevents Gram-negative bacterial lipopolysaccharide-induced vascular permeability. *Blood*.

[31] Seynhaeve A. L. B., Vermeulen C. E., Eggermont A. M. M., ten Hagen T. L. M. (2006). Cytokines and vascular permeability: an in vitro study on human endothelial cells in relation to tumor necrosis factor-*α*-primed peripheral blood mononuclear cells. *Cell Biochemistry and Biophysics*.

[32] Cardoso F. L., Kittel Á., Veszelka S. (2012). Exposure to lipopolysaccharide and/or unconjugated bilirubin impair the integrity and function of brain microvascular endothelial cells. *PLoS ONE*.

[33] Lopez-Ramirez M. A., Fischer R., Torres-Badillo C. C. (2012). Role of caspases in cytokine-induced barrier breakdown in human brain endothelial cells. *Journal of Immunology*.

[34] Abdullah Z., Bayraktutan U. (2014). NADPH oxidase mediates TNF-*α*-evoked in vitro brain barrier dysfunction: roles of apoptosis and time. *Molecular and Cellular Neuroscience*.

[35] Bechelli J., Smalley C., Milhano N., Walker D. H., Fang R. (2015). Rickettsia massiliae and rickettsia conorii israeli spotted fever strain differentially regulate endothelial cell responses. *PLoS ONE*.

[36] Yang Y., Chen Q.-H., Liu A.-R., Xu X.-P., Han J.-B., Qiu H.-B. (2015). Synergism of MSC-secreted HGF and VEGF in stabilising endothelial barrier function upon lipopolysaccharide stimulation via the Rac1 pathway. *Stem Cell Research & Therapy*.

[37] Wagner N.-M., Van Aken C., Butschkau A. (2016). Procalcitonin impairs endothelial cell function and viability. *Anesthesia & Analgesia*.

[38] McDonnell C. J., Garciarena C. D., Watkin R. L. (2016). Inhibition of major integrin *α*_V_*β*_3_ reduces Staphylococcus aureus attachment to sheared human endothelial cells. *Journal of Thrombosis and Haemostasis*.

[39] Zhu X., Zou Y., Wang B. (2016). Blockade of CXC chemokine receptor 3 on endothelial cells protects against sepsis-induced acute lung injury. *Journal of Surgical Research*.

[40] Wang J. H., Redmond H. P., Watson R. W. G., Bouchier-Hayes D. (1997). Induction of human endothelial cell apoptosis requires both heat shock and oxidative stress responses. *American Journal of Physiology—Cell Physiology*.

[41] Polunovsky V. A., Wendt C. H., Ingbar D. H., Peterson M. S., Bitterman P. B. (1994). Induction of endothelial cell apoptosis by TNF*α*: modulation by inhibitors of protein synthesis. *Experimental Cell Research*.

[42] Liu Z.-H., Striker G. E., Stetler-Stevenson M., Fukushima P., Patel A., Striker L. J. (1996). TNF-alpha and IL-1 alpha induce mannose receptors and apoptosis in glomerular mesangial but not endothelial cells. *American Journal of Physiology—Cell Physiology*.

[43] Arpino V., Mehta S., Wang L. (2016). Tissue inhibitor of metalloproteinases 3-dependent microvascular endothelial cell barrier function is disrupted under septic conditions. *American Journal of Physiology—Heart and Circulatory Physiology*.

[44] Hotchkiss R. S., Tinsley K. W., Swanson P. E., Karl I. E. (2002). Endothelial cell apoptosis in sepsis. *Critical Care Medicine*.

[45] Subbanna S., Shivakumar M., Umapathy N. S. (2013). G9a-mediated histone methylation regulates ethanol-induced neurodegeneration in the neonatal mouse brain. *Neurobiology of Disease*.

[46] Spanos A., Jhanji S., Vivian-Smith A., Harris T., Pearse R. M. (2010). Early microvascular changes in sepsis and severe sepsis. *Shock*.

[47] De Backer D., Orbegozo Cortes D., Donadello K., Vincent J.-L. (2014). Pathophysiology of microcirculatory dysfunction and the pathogenesis of septic shock. *Virulence*.

[48] Endo S., Inada K., Nakae H. (1995). Blood levels of endothelin-1 and thrombomodulin in patients with disseminated intravascular coagulation and sepsis. *Research Communications in Molecular Pathology and Pharmacology*.

[49] Cines D. B., Pollak E. S., Buck C. A. (1998). Endothelial cells in physiology and in the pathophysiology of vascular disorders. *Blood*.

[50] Mutunga M., Fulton B., Bullock R. (2001). Circulating endothelial cells in patients with septic shock. *American Journal of Respiratory and Critical Care Medicine*.

[51] Reinhart K., Bayer O., Brunkhorst F., Meisner M. (2002). Markers of endothelial damage in organ dysfunction and sepsis. *Critical Care Medicine*.

[52] Ware L. B., Eisner M. D., Thompson B. T., Parsons P. E., Matthay M. A. (2004). Significance of Von Willebrand factor in septic and nonseptic patients with acute lung injury. *American Journal of Respiratory and Critical Care Medicine*.

[53] Shapiro N. I., Aird W. C. (2011). Sepsis and the broken endothelium. *Critical Care*.

[54] Skibsted S., Jones A. E., Puskarich M. A. (2013). Biomarkers of endothelial cell activation in early sepsis. *Shock*.

[55] Nuckton T. J., Alonso J. A., Kallet R. H. (2002). Pulmonary dead-space fraction as a risk factor for death in the acute respiratory distress syndrome. *The New England Journal of Medicine*.

[56] Bull T. M., Clark B., McFann K., Moss M. (2010). Pulmonary vascular dysfunction is associated with poor outcomes in patients with acute lung injury. *American Journal of Respiratory and Critical Care Medicine*.

[57] Agrawal A., Matthay M. A., Kangelaris K. N. (2013). Plasma angiopoietin-2 predicts the onset of acute lung injury in critically ill patients. *American Journal of Respiratory and Critical Care Medicine*.

[58] Calfee C. S., Gallagher D., Abbott J., Thompson B. T., Matthay M. A. (2012). Plasma angiopoietin-2 in clinical acute lung injury. *Critical Care Medicine*.

[59] Sapru A., Calfee C. S., Liu K. D. (2015). Plasma soluble thrombomodulin levels are associated with mortality in the acute respiratory distress syndrome. *Intensive Care Medicine*.

[60] Terpstra M. L., Aman J., Van Nieuw Amerongen G. P., Groeneveld A. B. J. (2014). Plasma biomarkers for acute respiratory distress syndrome: a systematic review and Meta-Analysis. *Critical Care Medicine*.

[61] Matthay M. A., Ware L. B., Zimmerman G. A. (2012). The acute respiratory distress syndrome. *Journal of Clinical Investigation*.

[62] Ware L. B., Matthay M. A. (2000). The acute respiratory distress syndrome. *The New England Journal of Medicine*.

[63] Kuckleburg C. J., Tiwari R., Czuprynski C. J. (2008). Endothelial cell apoptosis induced by bacteria-activated platelets requires caspase-8 and -9 and generation of reactive oxygen species. *Thrombosis and Haemostasis*.

[64] Meguro T., Chen B., Parent A. D., Zhang J. H. (2001). Caspase inhibitors attenuate oxyhemoglobin-induced apoptosis in endothelial cells. *Stroke*.

[65] Sagoo P., Chan G., Larkin D. F. P., George A. J. T. (2004). Inflammatory cytokines induce apoptosis of corneal endothelium through nitric oxide. *Investigative Ophthalmology & Visual Science*.

[66] Shivanna M., Rajashekhar G., Srinivas S. P. (2010). Barrier dysfunction of the corneal endothelium in response to TNF-*α*: role of p38 MAP kinase. *Investigative Ophthalmology and Visual Science*.

[67] Zetter B. R. (1981). The endothelial cells of large and small blood vessels. *Diabetes*.

[68] Shelton J. L., Wang L., Cepinskas G. (2006). Albumin leak across human pulmonary microvascular vs. umbilical vein endothelial cells under septic conditions. *Microvascular Research*.

[69] Gerritsen M. E. (1987). Functional heterogeneity of vascular endothelial cells. *Biochemical Pharmacology*.

[70] Glassberg M. K., Nolop K. B., Jackowski J. T., Abraham W. M., Wanner A., Ryan U. S. (1992). Microvascular and macrovascular endothelial cells produce different constrictor substances. *Journal of Applied Physiology*.

[71] Wang Q., Pfeiffer G. R., Stevens T., Doerschuk C. M. (2002). Lung microvascular and arterial endothelial cells differ in their responses to intercellular adhesion molecule-1 ligation. *American Journal of Respiratory and Critical Care Medicine*.

[72] Matsuda N., Yamamoto S., Takano K.-I. (2009). Silencing of Fas-associated death domain protects mice from septic lung inflammation and apoptosis. *American Journal of Respiratory and Critical Care Medicine*.

[73] Matsuda N., Teramae H., Yamamoto S., Takano K.-I., Takano Y., Hattori Y. (2010). Increased death receptor pathway of apoptotic signaling in septic mouse aorta: effect of systemic delivery of FADD siRNA. *American Journal of Physiology—Heart and Circulatory Physiology*.

[74] Matsuda N., Takano Y., Kageyama S.-I. (2007). Silencing of caspase-8 and caspase-3 by RNA interference prevents vascular endothelial cell injury in mice with endotoxic shock. *Cardiovascular Research*.

[75] Galluzzi L., Bravo-San Pedro J. M., Vitale I. (2015). Essential versus accessory aspects of cell death: recommendations of the NCCD 2015. *Cell Death & Differentiation*.

[76] Galluzzi L., Vitale I., Abrams J. M. (2012). Molecular definitions of cell death subroutines: recommendations of the Nomenclature Committee on Cell Death 2012. *Cell Death and Differentiation*.

[77] Matute-Bello G., Frevert C. W., Martin T. R. (2008). Animal models of acute lung injury. *American Journal of Physiology—Lung Cellular and Molecular Physiology*.

[78] Xu C., Wu X., Hack B. K., Bao L., Cunningham P. N. (2015). TNF causes changes in glomerular endothelial permeability and morphology through a Rho and myosin light chain kinase‐dependent mechanism. *Physiological Reports*.

[79] Wu M. H. (2005). Endothelial focal adhesions and barrier function. *The Journal of Physiology*.

[80] Burnham E. L., Taylor W. R., Quyyumi A. A., Rojas M., Brigham K. L., Moss M. (2005). Increased circulating endothelial progenitor cells are associated with survival in acute lung injury. *American Journal of Respiratory and Critical Care Medicine*.

[81] Yoder M. C. (2011). Progenitor cells in the pulmonary circulation. *Proceedings of the American Thoracic Society*.

[82] Curley G. F., Laffey J. G., Zhang H., Slutsky A. S. (2016). Biotrauma and ventilator-induced lung injury: clinical implications. *Chest*.

[83] Potter D. R., Damiano E. R. (2008). The hydrodynamically relevant endothelial cell glycocalyx observed in vivo is absent in vitro. *Circulation Research*.

[84] Townsley M. I. (2012). Structure and composition of pulmonary arteries, capillaries, and veins. *Comprehensive Physiology*.

[85] Yang Y., Schmidt E. P. (2013). The endothelial glycocalyx: an important regulator of the pulmonary vascular barrier. *Tissue Barriers*.

[86] Dente C. J., Steffes C. P., Speyer C., Tyburski J. G. (2001). Pericytes augment the capillary barrier in in vitro cocultures. *Journal of Surgical Research*.

[87] Edelman D. A., Jiang Y., Tyburski J., Wilson R. F., Steffes C. (2006). Pericytes and their role in microvasculature homeostasis. *Journal of Surgical Research*.

